# Differential screening identifies transcripts with depot-dependent expression in white adipose tissues

**DOI:** 10.1186/1471-2164-9-397

**Published:** 2008-08-22

**Authors:** Yu Wu, Ji Young Kim, Shengli Zhou, Cynthia M Smas

**Affiliations:** 1 Department of Biochemistry and Cancer Biology, University of Toledo Health Science Campus, Toledo, OH 43614, USA

## Abstract

**Background:**

The co-morbidities of obesity are tied to location of excess fat in the intra-abdominal as compared to subcutaneous white adipose tissue (WAT) depot. Genes distinctly expressed in WAT depots may impart depot-dependent physiological functions. To identify such genes, we prepared subtractive cDNA libraries from murine subcutaneous (SC) or intra-abdominal epididymal (EP) white adipocytes.

**Results:**

Differential screening and qPCR validation identified 7 transcripts with 2.5-fold or greater enrichment in EP *vs*. SC adipocytes. Boc, a component of the hedgehog signaling pathway demonstrated highest enrichment (~12-fold) in EP adipocytes. We also identified a dramatic enrichment in SC adipocytes *vs*. EP adipocytes and in SC WAT *vs*. EP WAT for transcript(s) for the major urinary proteins (Mups), small secreted proteins with pheromone functions that are members of the lipocalin family. Expression of Boc and Mup transcript was further assessed in murine tissues, adipogenesis models, and obesity. qPCR analysis reveals that EP WAT is a major site of expression of Boc transcript. Furthermore, Boc transcript expression decreased in obese EP WAT with a concomitant upregulation of Boc transcript in the obese SC WAT depot. Assessment of the Boc binding partner Cdon in adipose tissue and cell fractions thereof, revealed transcript expression similar to Boc; suggestive of a role for the Boc-Cdon axis in WAT depot function. Mup transcripts were predominantly expressed in liver and in the SC and RP WAT depots and increased several thousand-fold during differentiation of primary murine preadipocytes to adipocytes. Mup transcripts were also markedly reduced in SC WAT and liver of *ob/ob *genetically obese mice compared to wild type.

**Conclusion:**

Further assessment of WAT depot-enriched transcripts may uncover distinctions in WAT depot gene expression that illuminate the physiological impact of regional adiposity.

## Background

In addition to its role in energy storage and mobilization, white adipose tissue (WAT) is an important endocrine organ that synthesizes and secretes various hormones and adipokines, a number of which impact systemic energy balance [1-4]. Various studies in humans and rodents have illuminated distinctions in the physiology, lipolytic response, gene expression and other aspects of adipocytes present in different WAT depots [5-20]. These observations have led to the suggestion that individual WAT adipose depots are best regarded as separate "miniorgans" [10]. These distinctions, and their molecular underpinnings, are gaining in importance with the realization that it is the anatomical location of excess adipose tissue that appears to underlie the health impact of obesity, and that interventions targeting reduction of intra-abdominal fat mass can effectively combat obesity-related diseases [15,21-25]. Several recent studies have sought to identify gene expression distinctions among preadipocytes or adipocytes of different WAT adipose depots [19,20]. However, a complete and defining picture of WAT depot dependent gene expression, as well as the underlying regulatory events governing depot-dependent gene expression, is yet to be identified.

In order to identify WAT depot-enriched transcripts, we undertook preparation and screening of murine suppressive subtractive hybridization (SSH) cDNA libraries enriched for genes expressed in either SC or EP murine adipocytes. Our studies reveal that transcripts for Mups, major urinary protein members of the lipocalin superfamily with pheromone function, exhibit a surprisingly distinctive pattern of transcript expression in WAT depots with dramatic upregulation noted for subcutaneous (SC) WAT and retroperitoneal (RP) WAT *vs*. the epididymal (EP) intra-abdominal WAT depot. SSH screening also identified 7 transcripts with enriched expression in EP adipocytes *vs*. SC adipocytes. Of these, Boc, an immunoglobulin superfamily member that functions in the hedgehog signaling network, exhibited the highest degree of differential expression.

## Methods

### Animal use and cellular fractionation of murine adipose tissues

All animal treatments were conducted with the approval of the University of Toledo Health Science Campus Institutional Animal Care and Use Committee. Mice were purchased from The Jackson Laboratory. For Northern blot and qPCR analyses of murine tissues, including in distinct adipose depots, 8-wk old C57Bl/6J male mice were utilized. For studies of gene expression in obese *vs*. wild type mice, we used 8-wk old male mice that were *ob/ob *homozygous on a C57Bl/6J background (strain designation, B6.V-Lep^*ob/ob*^) or wild type C57Bl/6J mice generated from breeding of *ob*/+ heterozygotes. Fractionation of whole adipose tissue into adipocyte fraction (AF) and stromal-vascular fraction (SVF) was *via *collagenase digestion and differential centrifugation, as previously described [26-28], starting with pooled tissue of 6 mice. Resultant cell fractions were either used directly for RNA preparation, or in the case of primary culture differentiation studies (see below) SVF cells were plated and cultured in DMEM with 10% FCS.

### RNA preparation and transcript analysis

For analysis of transcript expression in murine adipose depots, the SC, retroperitoneal (RP), and EP WAT and interscapular brown adipose tissue (BAT) were dissected from four individual 8-wk old male C57Bl/6J mice. Tissues were minced, frozen in liquid nitrogen, and homogenized in TriZol reagent using a polytron. Other murine tissues were similarly processed. Total RNA was purified using TriZol reagent according to manufacturer's instruction (Invitrogen Corp.). For Northern blot analysis, 5 μg of total RNA was fractionated in 1% agarose-formaldehyde gels in MOPS buffer and transferred to Hybond-N membrane (GE Healthcare, Piscataway, NJ). Blots were hybridized in ExpressHyb solution (BD Biosciences) for 1 h at 65°C with the indicated randomly primed ^32^P-dATP-labeled cDNA insert probes. After washing for 20 min at 65°C with 1% SDS in 1× SSC and for 30 min at 65°C with 0.1% SDS in 0.1× SSC, membranes were exposed at -80°C to Kodak BioMax film with a Kodak BioMax intensifying screen. Northern blot analysis was conducted in duplicate and representative data is shown. All lanes shown as a single autoradiographic image were run on the same blot, however in some instances lanes may have been reordered or removed for economy and/or clarity of presentation.

For reverse-transcription and quantitative analysis of gene expression by qPCR, total RNA was subject to purification using an RNeasy kit with DNase I treatment (Qiagen Corp., Valencia, CA) and cDNA synthesized with SuperScript II RNase H(-) reverse transcriptase (Invitrogen Corp.) using an oligo (dT)-22-mer primer. SYBR green-based qPCR was conducted with an ABI 7500 Real-Time PCR System (Applied Biosystems, Foster City, CA). Reaction conditions were 1× SYBR Green PCR Master Mix (Applied Biosystems), 100 nM each forward and reverse primers, and 10 ng of cDNA. PCR was carried out over 40 cycles of 95°C for 15 sec, 60°C for 30 sec, and 72°C for 34 sec with an initial cycle of 50°C for 2 min and 95°C for 10 min. All primers were designed to span intron locations and qPCR assays were conducted in triplicate. Primer sequences used were: Gapdh, 5'-GGCAAATTCAACGGCACAG-3' and 5'-CGGAGATGATGACCCTTTTGG-3'; 36B4 (gene name: acidic ribosomal phosphoprotein P0/Arbp), 5'-GAGACTGAGTACACCTTCCCAC-3' and 5'-ATGCAGATGGATCAGCCAGG-3'; Boc, 5'-AAACAGCAGTGAGGCGAAC-3' and 5'-CACTTGGCAGGAGTCAGAAC-3'; Cdon, 5'-TAACATACTGAGCCCCCCACAG-3' and 5'-CACTACCATCGTCCAGCTTTCG-3'; Mup1, 5'-AAGAACAAGCAAAGGGGCTGGG-3' and 5'-ACACAGCAGCAGCAGCATCTTC-3'; Mup1/2, 5'-ACTGACCCTAGTCTGTGTCC-3' and 5'-AGCCTTTTCTGTTTTGTCAGC-3'; Tuba1, 5'-GCAGCCGCGAAGCAGCAAC-3' and 5'-CCATGTTCCAGGCAGTAGAGCT-3'; Serping1, 5'-GTCCAAATTCCTGCCCACTTAC-3' and 5'-TCAGTTCCAGCACTGTCTCG-3'; Timp4, 5'-TGGAAAAGTCTTCATCCATCTG-3' and 5'-GGTACATGGCACTGCATAG-3'; Col4a2, 5'-ACACTGTGGACTTACCAGG-3' and 5'-CCAGGAAATCCAATGTCACC; H6pd, 5'-AGAAGAGCAGTGCCATCCTG-3' and 5'-TCGATGTGGACAAGGACACC-3'; Fos, 5'-CCCCAAACTTCGACCATGATG-3' and 5'-AGTTGGCACTAGAGACGGAC-3'.

Specific transcript expression was normalized against respective Gapdh and 36B4 signals and fold differences calculated. Detection Gapdh or the 36B4 signals between compared sample sets rarely differed by more than one or two cycles. Graphical data is presented for transcript expression levels calculated by correction to either Gapdh or 36B4 internal control transcripts; these values are readily apparent in the respective graph. However, for clarity of presentation of data in the text, fold differences are presented as the average of the Gapdh and the 36B4 corrected values. The p values stated in the text are applicable to data generated with either correction for the Gapdh or 36B4 internal controls and only those data that meet the criteria of statistically significant differential transcript expression upon correction with both Gapdh and 36B4 are discussed in the text.

### Suppression subtractive hybridization (SSH) and differential screening of SSH cDNA libraries

We employed the SSH method to generate subtractive cDNA libraries for transcripts enriched in SC WAT adipocytes or EP WAT adipocytes. SC WAT and EP WAT were collected from six 8-wk old male C57Bl/6J mice. The SC WAT and EP WAT from individual animals was pooled and fractionated into adipocyte and SVF cell fractions *via *collagenase digestion as previously described [26-28] and total adipocyte RNA isolated using TriZol. A PCR-Select cDNA Subtraction Kit (BD Biosciences, Palo Alto, CA) was employed according to manufacturer instructions, to generate an SC adipocyte and an EP adipocyte SSH library, starting from 5 μg of total RNA. The resultant pools of PCR products consisting of double stranded cDNAs were subcloned into the pGEM-T vector (Promega) and transformed into DH5α *E. coli *to create SC SSH and EP SSH plasmid-based libraries as *E. coli *stocks.

The SC adipocyte SSH cDNA library and EP adipocyte SSH cDNA library were screened by differential hybridization of high-density nylon cDNA arrays. Arrays were prepared by robotic spotting of PCR-amplified inserts of SSH library clones *via *contract arrangement with the German Resource Center for Genome Research (RZPD, ). The SSH library we prepared was sent to RZPD as glycerol stock; RZPD plated the library and robotically picked individual colonies and PCR amplified clone inserts using PCR primers for sequences flanking the pCR2.1-TOPO vector (Invitrogen Corp.) multi-cloning site. PCR-amplified inserts were spotted in duplicate from the SSH SC adipocyte and the SSH EP adipocyte libraries to generate high density nylon arrays, which were returned to us for differential screening. For this, membranes were prehybridized at 65°C for 1 h in ExpressHyb solution containing 20× SSC and 50 μg of salmon sperm DNA and hybridized overnight at 65°C using ^33^P-dATP-labeled reverse-transcribed probes synthesized from 8 μg of EP adipocyte total RNA or SC adipocyte total RNA. Following hybridization, membranes were washed four times in 2× SSC/0.5% SDS at 65°C for 20 min each, followed by two 20 min washes in 0.2× SSC/0.5% SDS at 65°C, after which they were exposed at -80°C to Kodak BioMax film with a Kodak BioMax intensifying screen. Signals were analyzed visually and candidate differentially expressed cDNAs were sequenced. Both the fractionated material used to generate the SSH library and that used to screen the library was validated for fractionation into adipocyte and stromal fractions based on expression of marker transcripts for these two fractions. The adipocyte fraction was determined to be positive for transcript expression of SCD1 and negative for TSC-36, a marker we have identified for the SVF fraction of adipose tissue [27]; the opposite pattern was observed for the SVF. The adipocyte fraction was also negative for macrophages and endothelial cells based on the lack of signal for emr1/F80 and von Willebrand factor transcripts, respectively.

### Adipocyte differentiation

3T3-L1 cells (American Type Culture Collection, Manassas, VA) were propagated in DMEM supplemented with 10% calf serum. For differentiation, 3T3-L1 cells were treated at two days post-confluence with DMEM supplemented with 10% FCS in the presence of the adipogenic inducers 0.5 mM methylisobutylxanthine (MIX) and 1 μM dexamethasone for 48 h. Adipogenic agents were then removed, and growth of cultures continued in DMEM containing 10% FCS. At five days post-induction of differentiation, adipocyte conversion had occurred in approximately 90% of the cells, as judged by lipid accumulation and cell morphology.

Murine primary preadipocyte SVF cultures were prepared from SC WAT of 8-wk old C57Bl/6J male mice, as described under *Animal Use and Cellular Fractionation of Murine Adipose Tissues*, above. Cells were propagated in DMEM supplemented with 10% FCS. For differentiation, cultures were treated at two days post-confluence with DMEM supplemented with 10% FCS in the presence of the adipogenic inducers 0.5 mM MIX, 1 μM dexamethasone, 0.2 mM indomethacin, and 170 nM insulin for 72 h. Adipogenic agents were then removed and growth of cultures continued in DMEM containing 10% FCS. Primary cell differentiation was analyzed on two sets of cultures with essentially the same results.

## Results

### Differential enrichment of Boc and Cdon transcripts in EP adipocytes and EP WAT

**Figure 1:**
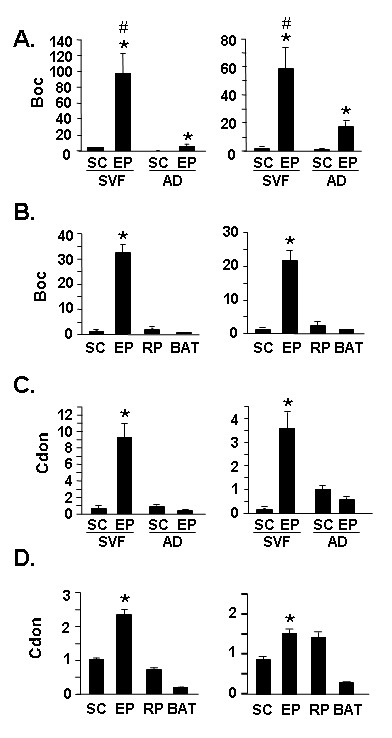
**Expression of transcripts for hedgehog signaling components Boc and Cdon in adipose depots**. **A**. qPCR assessment of transcript levels in SVF and AD fractions of SC and EP WAT using the Boc primer set. **B**. qPCR assessment of Boc transcript in whole SC, EP, RP or BAT adipose tissue. **C**. qPCR assessment of transcript levels in SVF and AD of SC and EP WAT using the Cdon primer set. **D**. qPCR assessment of Cdon transcript in whole SC, EP, RP or BAT adipose tissue. For A-D, the left panels show data corrected against Gapdh and the right panels show data corrected against 36B4 as internal control for qPCR analysis; values stated in the text are the average of the Gapdh-corrected and 36B4-corrected data for each comparison. In A and B, the level in SC AD was set to a value of 1. SVF, stromal vascular fraction; AD, adipocyte fraction. In B and D, the level in SC WAT was set to a value of 1. For A, * indicates p < 0.001 for EP SVF *vs*. SC SVF and for EP AD *vs*. SC AD and # indicates p < 0.001 for EP SVF *vs*. all others (both panels). For B, * indicates p < 0.001 for EP *vs*. all others. For C, * indicates p < 0.001 for EP SVF *vs*. all others (both panels). For D, * indicates p < 0.001 for EP *vs*. all others (left panel) and EP *vs*. SC and BAT (right panel).

Given the relationship between regional adiposity and health morbidities we set out to identify transcripts that evidenced enriched expression in specific WAT depots by creating and screening SSH cDNA libraries designed to be enriched for transcripts present in EP or SC adipocytes. Genes that were identified in our SSH screening showing differential transcript expression in EP adipocytes *vs*. SC adipocytes were validated by qPCR. Of these, Boc demonstrated the greatest degree of transcript enrichment in EP adipocytes *vs*. SC adipocytes. Boc is a binding partner for Cdon, also known as cell adhesion molecule-related/down-regulated by oncogene [29,30]. Boc and Cdon are both immunoglobulin superfamily members that are components of the hedgehog signaling pathway [29,30]. qPCR analysis in Figure 1A shows that Boc transcript is enriched an average 12-fold (p < 0.001) in EP adipocytes *vs*. SC adipocyte and an average 32-fold (p < 0.001) in the EP SV cell fraction *vs*. SC SV cell fraction. When intact adipose tissue is assessed, Boc transcript shows an average 27-fold (p < 0.001) enrichment in EP WAT *vs*. SC WAT (Figure 1B). Boc transcript level similar to that for SC is noted in RP WAT and BAT. These data suggest that it is not solely the adipocytes in EP adipose tissue that are enriched for Boc transcript, but that enrichment is also found for cell type(s) in the SV fraction. We next examined whether a similar pattern of expression might be noted for transcript for the Boc binding protein Cdon. We find that Cdon transcript is also enriched in EP WAT *vs*. SC WAT, although this is only noted for the SV fraction (Figure 1C) or intact WAT (Figure 1D), and not for isolated adipocytes (Figure 1C). As Boc and Cdon transcripts were both detected in adipocytes we also tested whether their transcript expression level was altered in adipogenesis by assessing levels in 3T3-L1 cells, a well characterized model of *in vitro *adipocyte differentiation, and in the *in vitro *differentiation of primary preadipocytes to adipocytes. In both cases, the levels of Boc and Cdon transcripts were not appreciably different in preadipocytes *vs. in vitro *differentiated adipocytes (data not shown).

**Figure 2:**
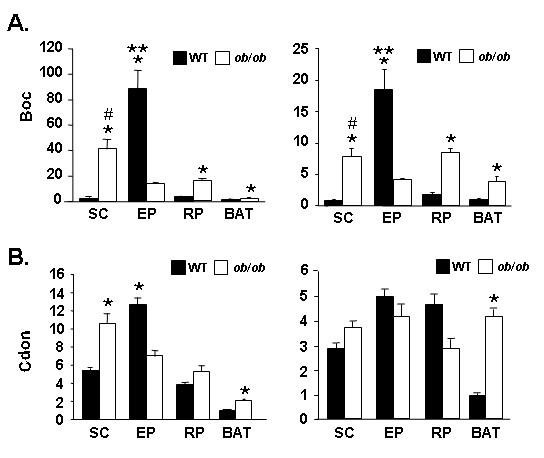
**Expression of Boc and Cdon transcript in wild type and *ob/ob *tissues**. qPCR assessment of transcript level in wild type C57Bl/6J (WT) and *ob/ob *SC WAT, EP WAT, RP WAT and BAT depots using the Boc primer set (A) and the Cdon primer set (B). For A and B, the left panels show data corrected against Gapdh and the right panels show data corrected against 36B4 as internal control for qPCR analysis; values stated in the text are the average of the Gapdh-corrected and 36B4-corrected data for each comparison. The level in WT BAT was set to a value of 1. For A, * indicates p < 0.001 for comparisons of WT *vs. ob/ob *samples for each of SC, EP, RP and BAT and # indicates p < 0.005 for *ob/ob *SC *vs*. all other *ob/ob *samples, and **, P < 0.005 for WT EP *vs*. all other samples (both panels). For B, * indicates p < 0.001 for WT *vs. ob/ob *BAT WT (both panels) and for WT *vs. ob/ob *for SC and EP depots left panel only.

**Figure 3:**
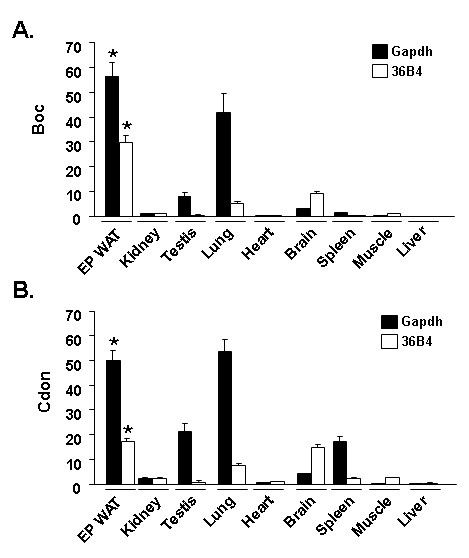
**Tissue distribution of Boc and Cdon transcript expression**. qPCR analysis using the Boc (A) or the Cdon (B) primer set. For A and B, data was corrected against Gapdh (black fill) and or 36B4 (white fill) as internal control for qPCR analysis; values stated in the text are the average of the Gapdh-corrected and 36B4-corrected data for each comparison. For A, * indicates p < 0.001 for EP WAT compared with all tissues except the Gapdh-corrected value for lung. For B, * indicates p < 0.001 for EP WAT compared with all tissues except the Gapdh-corrected value for lung and the 36B4-corrected value for brain. For A and B, the level in kidney was set to a value of 1.

Since neither the expression of transcripts for Boc nor Cdon had been previously assessed in adipose tissue, but the hedgehog pathway plays a role in fat formation [31-33], we next determined transcript expression in wild type and obese (*ob/ob*) murine adipose tissues by qPCR (Figure 2). We find that alteration of Boc transcript level occurs in each of the 4 depots examined, with *ob/ob *mice showing upregulation of Boc transcript in SC, RP and BAT, and downregulation in EP (Figure 2A). Thus we find that for *ob/ob*, Boc transcript depot-dependence in SC *vs*. EP WAT is opposite to that observed for wild type WAT depots, namely SC *ob/ob *WAT shows the highest degree of expression of Boc transcript is an average 2.5 times (p < 0.001) that found in *ob/ob *EP WAT. For Cdon, upregulation of transcript is noted in *ob/ob vs*. WT for the SC and BAT depots of an average 1.6-fold (p < 0.01) and 4-fold (p < 0.001), respectively (Figure 2B). To our knowledge, only limited assessment of murine tissue expression patterns have been reported for Boc and Cdon transcript, and studies assessing expression in adipose tissue *vs*. other tissues have not been carried out. To determine if EP WAT is a dominant site of expression of these genes *in vivo*, we used qPCR to compare Boc and Cdon transcript levels in kidney, testis, lung, heart, brain, spleen, muscle and liver with that for EP WAT (Figure 3). EP WAT was chosen for this comparison, since it expressed the highest level of Boc transcript of the four adipose depots we had examined (Figure 1). Of the nine murine tissues examined, EP WAT was the site of highest expression of Boc transcript (Figure 3A) and it was among the highest site for expression of Cdon transcript (Figure 3B). Future work from this laboratory will address the functional role Boc and Cdon may play in distinct WAT depots.

Data for the other 6 genes that we identified as enriched in EP *vs*. SC adipocytes are presented in Additional file 1. However, these do not meet a criteria of statistically significant (p < 0.01) of EP *vs*. SC depot enriched expression for isolated adipocytes as well as in whole EP *vs*. SC WAT. For example, Fos is only minimally expressed in whole WAT of either the SC or EP depot, but its level is dramatically elevated in response to the isolation procedure *per se*, as has been described for a number of genes [34].

### Differential screening reveals highly enriched expression of Mup transcripts in the SC WAT depot

Our analyses of differential hybridization of SSH SC library clones revealed that approximately 50 of the cDNA clones with increased expression in SC adipocytes *vs*. EP adipocytes contained sequences corresponding to major urinary protein. Major urinary proteins (Mups) are small acidic molecules with molecular mass of ~19 kDa that belong to the lipocalin superfamily [35]. Lipocalins share a novel conserved calyx-shaped β barrel structure [36-44] and proteins in this family are proposed to serve dual molecular functions in the transport of lipophilic molecules and in the regulation of cell homeostasis [45]. Mups exist as a complex array of protein isoforms generated from the multigene Mup gene family present on murine chromosome 4 [46]; they are present in serum and are the major protein constituent of urine in the mouse [47]. The Mup gene family includes functional genes, pseudogenes and silent genes [48-57]; our recent analysis of the Ensembl database  indicated 44 gene sequences in this family. Only a handful of Mup genes and gene products have been characterized in any detail, mainly Mup1 – Mup5 [58]. The most recent studies of Mup transcripts expression were conducted roughly two decades ago [48-57], when the extent of gene sequence similarity and complexity of the Mup gene family was likely not fully appreciated. Mup gene expression has not been reported to any extent in the intervening time period. In retrospect, it is unclear if single specific Mup transcript species, or rather sets of Mup transcripts, were truly under study in earlier reports.

**Figure 4:**
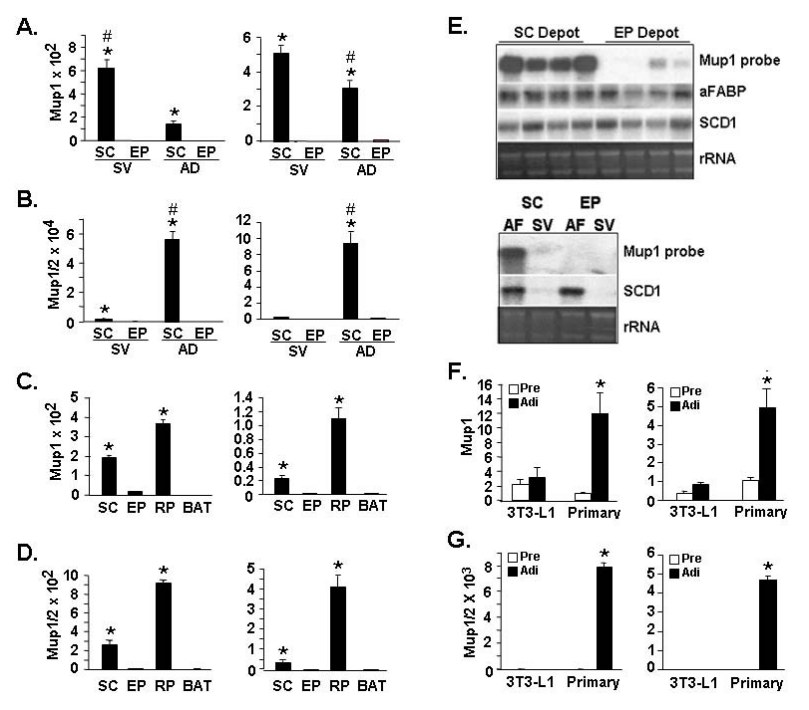
**Differential enrichment of Mup transcripts in the SC WAT depot**. **A**. qPCR assessment of transcript levels in SVF and AD fractions of SC and EP WAT using the Mup1 primer set. SVF, stromal vascular fraction; AD, adipocyte fraction. **B**. qPCR assessment of transcript levels in SVF and AD of SC and EP WAT using the Mup1/2 primer set. **C**. qPCR assessment of whole SC, EP, RP or BAT adipose tissue using the Mup1 primer set. **D**. qPCR assessment of whole SC, EP, RP or BAT adipose tissue using the Mup1/2 primer set. For A the EP SVF signal level was set to a value of 1 and for B the EP AF signal was set to 1. For A, * indicates p < 0.001 for SC SVF *vs*. EP SVF and for SC AD *vs*. EP AD, and # indicates p < 0.01 for SC SVF *vs*. all others. For B, * indicates p < 0.001 for SC SVF *vs*. EP SVF and for SC AD *vs*. EP AD, and # indicates p < 0.001 for SC AD *vs*. all others. For C and D the signal level in BAT was set to 1 and * indicates p < 0.001 for SC or RP compared with EP and with BAT. **E**. Upper panel shows Northern blot analysis of depot-dependent gene expression in either SC WAT or EP WAT depots of four individual male C57Bl/6J mice using ^32^P dATP-labeled Mup1, aFABP or SCD1 hybridization probes. Lanes 1–4 and lanes 5–8 represent SC and EP WAT tissue from mouse #1, #2, #3 and #4, respectively. Lower panel shows Northern blot analysis of fractionated SC and EP WAT. SV, stromal vascular fraction; AF, adipocyte fraction. Ethidium bromide staining of rRNA is shown as gel loading control. **F**. and **G**. qPCR assessment for 3T3-L1 or primary cultures of preadipocytes (Pre) and adipocytes (Adi) using the Mup1 (F) or the Mup1/2 (G) primer sets. The level of transcript expression in primary preadipocytes was set to a value of 1. For F and G, * indicates p < 0.001 for primary adipocytes *vs*. all others. For A-D, F and G, the left panels show data corrected against Gapdh and the right panels show data corrected against 36B4 as internal control for qPCR analysis; values stated in the text are the average of the Gapdh and 36B4-corrected data for each comparison.

An extremely high degree of identity is found in the sequences of various Mup transcripts with difference in sequence among members of the Mup multigene family often occurring as only scattered single or several base variations [59]. As such, the nearly identical nucleotide sequences of a number of different Mup gene products, particularly those with a high degree of identity with Mup1 and Mup2, render a number of Mup transcripts virtually indistinguishable by either Northern blot or PCR-based methods. Nonetheless, in an attempt to more precisely investigate the nature of differential Mup transcript expression, we designed PCR primer pairs that would be predicted to distinguish the gene products of Mup1 and of 4 Mup1-related genes from that for other Mup sequences; Mup1 and a subset of Mup1-related genes contain a unique region of ~40 nucleotides at the 5' end of the respective transcript(s). The qPCR data employing the Mup1 PCR primer set on fractionated WAT tissues is shown in Figure 4A and indicates higher Mup transcript expression in the SC depot compared to the EP depot. The signal detected with the Mup1 primer set is an average 560 times higher (p < 0.001 in SC SV fraction than in the EP SV fraction) and an average 100-fold higher (p < 0.001) in the SC AD fraction than in the EP AD fraction. To further discern adipose tissue expression of Mup transcripts, we utilized a second primer set, designated Mup1/2. Based on our assessment of the Mup multigene family, the Mup1/2 primer set is predicted to detect the same set of Mup transcripts detected by the Mup1 primer set, and 11 additional Mup transcripts, including Mup2. Compared with the signal from the Mup1 primer set, wherein transcripts are highly enriched in the SC SV fraction, transcripts detected with the Mup1/2 primer set are greatly enriched in the SC AD fraction. Here, the SC AD fraction signal is an average 70-fold higher (p < 0.001) than the SC SV fraction. Enrichment of signal in the SC *vs*. the EP depot is also evident. The signal in the SC AD fraction is an average 880-fold higher (p < 0.001) than in the EP AD fraction and the SC SV fraction signal is an average 1100-fold higher (p < 0.001) than the EP SV fraction.

When levels in whole adipose tissue are examined with the Mup1 primer set (Figure 4C), compared to EP WAT, an average 13-fold higher (p < 0.001) level of transcript is noted for SC WAT and an average 48-fold higher (p < 0.001) for the RP depot. Interestingly, although RP WAT is also intra-abdominal in location, it nonetheless expresses Mup transcript(s) at an order of magnitude that is similar to that noted for the SC WAT depot. Figure 4D shows that, similar to our findings with the Mup1 primer set, the Mup1/2 primer set detects enrichment of Mup transcripts in SC WAT and RP WAT. However, here we find that compared with the EP WAT, an average 43-fold higher (p < 0.001) level of transcript expression is noted for SC WAT and an average 270-fold higher (p < 0.001) level of transcript expression for the RP WAT depot (p < 0.001). Although the overall pattern of Mup transcript expression noted with the Mup1 and Mup1/2 primer sets is similar, these data also suggest a greater degree of depot-differential Mup transcript expression is found within that population of transcripts detected with the Mup1/2 primer set. We also conducted Northern blot analysis on SC and EP WAT of four individual mice using the Mup1 sequence as probe and included hybridization for the adipocyte marker transcripts aFABP and SCD1 for comparison purposes. Due to the high degree of sequence similarity and transcript size among various Mups, this analysis would be predicted to examine a population of various Mup transcripts. The Northern blot in Figure 4E (top panel) indicates clearly higher expression of signals detected by the Mup1 probe in the SC WAT depot, with a dramatically lower signal for EP WAT. The lower panel of Figure 4E reveals that in fractionated SC and EP WAT, it is the SC adipocytes that show the highest expression of signals detected by the Mup1 probe.

Since our data illustrated enrichment of Mup transcript in adipocytes *vs*. SV fraction cells, wherein preadipocytes are found, we next examined whether upregulation of Mup transcripts accompanied adipogenic conversion. qPCR analysis with the Mup1 and Mup1/2 primer sets revealed low levels of transcript expression in 3T3-L1 preadipocytes that were not appreciably altered during their conversion to adipocytes (Figure 4F and 4G). Therefore we next tested the differentiation-dependent expression of Mup transcripts using primary preadipocyte cultures prepared directly from murine SC WAT, and which might therefore be more reflective of the *in vivo *setting. Use of the Mup1 primer set, shown in Figure 4F, indicates an average 8-fold (p < 0.001) increase occurs during adipogenesis of primary cultures. Use of the Mup1/2 primer set (Figure 4G), reveals an average 5200-fold (p < 0.001) increase in level of transcript(s) detected by this primer set.

### Dysregulation of Mup transcript expression in WAT of *ob/ob *genetically obese mice

**Figure 5:**
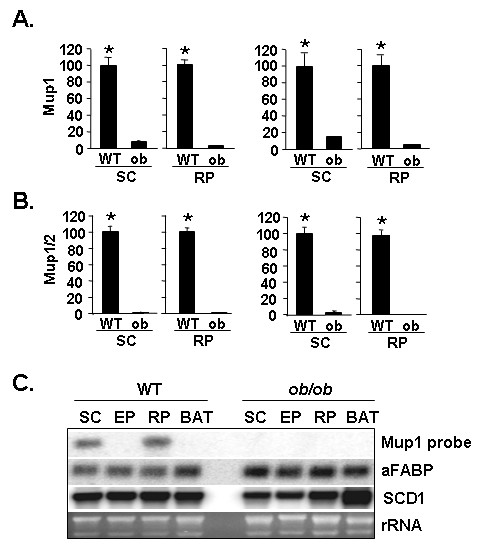
**Reduced expression of Mup transcript(s) in WAT of *ob/ob *mice. A**. qPCR assessment of transcript level in wild type (WT) and *ob/ob *(ob) SC WAT and RP WAT depots using the Mup1 primer set. **B**. qPCR assessment of transcript level in WT and ob SC WAT and RP WAT depots using the Mup1/2 primer set. For each graph, the signal in the respective WT tissue was set to a value of 100. For A and B the left panel shows data corrected against Gapdh and the right panel show data corrected against 36B4 as internal control for qPCR analysis; values stated in the text are the average of the Gapdh-corrected and 36B4-corrected data for each comparison. For A and B, * indicates p < 0.001 for WT SC *vs*. ob SC and for WT RP *vs*. ob RP. **C**. Northern blot analysis of 5 μg of total RNA from the indicated WAT depot or BAT from WT or *ob/ob *mice. Blot was hybridized to Mup1, aFABP or SCD1 ^32^P dATP-labeled probes. Ethidium bromide staining of rRNA is shown as a gel loading control.

To examine whether Mup transcript(s) expression was altered in obesity we utilized the *ob/ob *genetic model of murine obesity and compared expression with that of wild type mice. qPCR data obtained with the Mup1 and Mup1/2 primer sets are shown in Figures 5A and 5B. For the Mup1 primer set, compared with SC WAT and RP WAT from *ob/ob *mice, we find an average 10-fold (p < 0.001) higher transcript level in wild type SC WAT and an average 26-fold (p < 0.001) higher level for wild type RP WAT. For the Mup1/2 primer set, compared with SC WAT and RP WAT of *ob/ob *mice, we note an average 60-fold (p < 0.001) higher transcripts expression for wild type SC WAT and an average 230-fold (p < 0.001) higher level of transcripts for wild type RP WAT (p < 0.001). The Northern blot wherein the Mup1 sequence was used as a probe (Figure 5C) reveals a lack of Mup transcript signal in SC, EP, RP and BAT of WAT of *ob/ob *mice. Our findings indicate that Mup transcript(s) evidence differential expression not only across WAT depots, but also in respect to a well-established genetic model of murine obesity. That we find a differential degree of Mup transcript(s) enrichment in C57Bl/6J wild type and *ob/ob *mice when we use the Mup1 primer set *vs*. the Mup1/2 primer set suggests that within the Mup multigene family there are distinctions regarding the influence of obesity on the degree of differential expression of particular Mup transcript(s).

### Mup transcript expression in murine tissues

**Figure 6:**
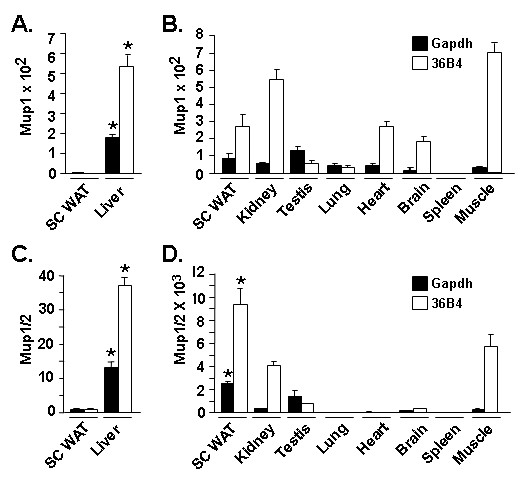
**qPCR analysis of tissue distribution of Mup transcript(s) expression**. qPCR analysis using the Mup1 primer set (A and B) or the Mup 1/2 primer set (C and D). For A and C the SC WAT level was set to a value of 1. For *B *and *D *the transcript level in spleen was set to a value of 1. For A – D, data was corrected against Gapdh (black fill) and or 36B4 (white fill) as internal control for qPCR analysis; values stated in the text are the average of the Gapdh-corrected and 36B4-corrected data for each comparison. For A and C, * indicates p < 0.001 for liver *vs*. SC WAT. For D, * indicates p < 0.05 for SC WAT *vs*. all others.

**Figure 7:**
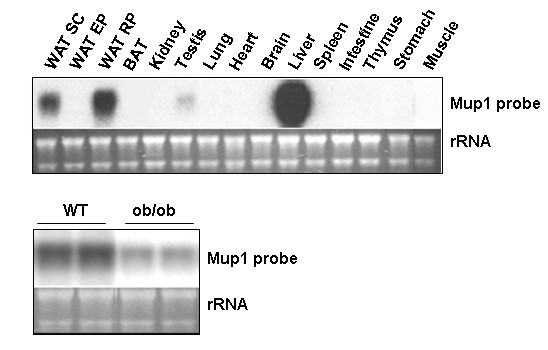
**Northern blot analysis of Mup transcript expression in murine tissues**. Northern blot analysis of a panel of C57Bl/6J murine tissues hybridized with a ^32^P dATP-labeled Mup1 probe. Lower panel shows Northern blot analysis of liver tissue from two wild type (WT) and two *ob/ob *mice. Ethidium bromide staining of rRNA is shown as a gel loading control.

Previous studies indicated that Mup transcript expression appeared particularly enriched in livers and also in other select tissues with secretory function; adipose tissue is now recognized as a secretory organ [1,60]. To examine the relative expression of Mup transcript(s) in adipose tissues *vs*. other murine tissues, we conducted qPCR and Northern blot analysis. SC WAT was chosen as a positive control for these comparisons since it expressed readily detected levels of Mup transcripts both by qPCR and Northern blot (Figures 4 and 5). Figure 6 shows qPCR analysis for the level of transcript(s) expression detected with the Mup1 (Figures 6A and 6B) or Mup1/2 primer set (Figures 6C and 6D). The Mup1 primer set (Figure 6A) detects an average 350-fold (p < 0.001) higher signal in liver *vs*. SC WAT and the Mup1/2 primer set (Figure 6C) an average 25-fold (p < 0.01) enrichment in liver compared to SC WAT. Figures 6B and 6D assess Mup transcript(s) expression in SC WAT and a panel of other murine tissues. For the Mup1 primer set (Figure 6B), a roughly similar level of expression is noted for SC WAT and all other tissues with the exception of spleen, which evidences minimal expression. In contrast, a different pattern of Mup transcript(s) expression is noted with the Mup1/2 primer set (Figure 6D), with SC WAT evidencing the highest expression. The differential nature of the signals obtained with the Mup1 and Mup1/2 primer sets suggests that among the multiple Mup transcript populations we detect herein, a number of individual Mup transcripts likely evidence distinctive patterns of tissue restricted expression. The Northern blot in the upper panel Figure 7 reveals that while liver tissue exhibits strongest signal detectable upon hybridization with the Mup1 probe, that of the tissues tested SC WAT is also a predominant site of expression of Mup transcripts. Since we note that the highest signal detected by the Mup1 probe are in liver, we also examined whether the level of these transcript was reduced in the liver of ob/ob, has we had previously found for SC and RP adipose tissue in such mice. The Northern blot in the lower panel of Figure 7 indicates a marked reduction of Mup transcripts in the liver of *ob/ob vs*. wild type mice.

## Discussion

The link between regional adipocyte burden and health morbidities is becoming increasingly apparent and thus it is key to reach beyond studies of adipogenesis *per se*, into studies that describe and explain gene expression in either the adipocyte and/or non-adipocyte cellular component of specific WAT depots. Such studies, however, are dependent on the discovery and validation of model genes that show a robust adipocyte depot-dependent phenotype. Our data herein and the work of others [19,20,61] have identified genes with differential pattern(s) of expression in regard to WAT depot expression. Kahn and colleagues recently used DNA microarray chips to assess transcript expression in adipocytes and SVF cells from murine SC WAT and EP WAT depots [19]. They identified 197 transcripts that met their criteria of differential expression for both adipocytes and SVF from SC WAT *vs*. EP WAT; the vast majority of the reported genes were altered less than 3-fold [19]. Interesting insights into the nature of WAT depot-dependent gene expression were, however, revealed in their further analysis of WAT depot-dependent expression of 12 embryonic development and pattern specification genes [19]. In these cases, the depot-dependence appeared cell autonomous in nature and was also observed for human WAT samples. Furthermore, the transcript levels of a subset of the 12 genes correlated with waist-to-hip ratio and/or body mass index, two established indices of human regional adiposity [19]. These workers also found differential expression of Mup transcripts in the SC WAT depot (74-fold higher in SC *vs*. EP); however as Mup transcript(s) were not among the small subset of developmental and/or patterning genes that were the focus of their study, its expression was not further validated or examined. Additionally, both our study and theirs observed increased expression of enrichment of tubulin alpha 1 transcript in the EP white adipose cells and tissue depot compared to that for SC [19]. It should also be kept in mind that these studies have examined transcript levels and whether the corresponding protein levels show differential expression remains to be determined. That we did not identify a larger set of differentially-expressed genes in common with the Kahn study [19], may be due to relative differences in sensitivity and/or comprehensiveness of the experimental methods employed. It is unlikely to be due to sample preparation as we have since assessed our RNA preparations for three depot-enriched transcripts identified by Kahn and coworkers [19] and found results similar to those they reported. Namely, levels of transcripts for Tbx15 and Shox2 were markedly higher in EP WAT than SC WAT and that for Phldb2 was higher in SC WAT than EP WAT (data not shown).

Uncovering gene expression distinctions across WAT depots have the potential to elucidate the underlying mechanism(s) for the development and/or function of specific adipose depots, and cell types therein, and their relation to disease. Comparison of the transcriptomes of visceral *vs*. subcutaneous adipocytes from WAT might ultimately provide therapeutic interventions that target visceral adipocytes while sparing white adipocytes in other WAT depots. While these types of gene expression studies are relatively straightforward, they also carry an important caveat. It has been clearly documented that the standard collagenase isolation procedure, which is necessary to separate whole adipose tissue into adipocyte and other cellular fractions, in itself, results in marked alterations in gene expression [34]. This is attributed in part to the impact of released cytokines, such as TNFα, and other factors from adipose tissue cell components on adipocyte and/or SVF cell gene expression [34]. Such concerns apply to our investigation as we produced and screened our subtracted libraries using the isolated adipocyte component of SC and EP WAT depots. However, we utilized both fractionated and whole adipose tissue samples for the detailed qPCR validation of depot-differential expression, and found that differential expression of Boc and Mup occurred both when isolated cell fractions and whole adipose tissue depots were assessed. On the other hand, the expression level of Fos transcript (see Additional file 1) is 1000 times higher in the fractionated cell samples *vs*. intact adipose tissue; Fos is therefore an example of a gene whose expression is dramatically altered due to the collagenase digestion protocol. Another concern that arises in regard to qPCR studies is that transcript expression is calculated relative to an internal control standard [62-64]; which by definition is expressed at a consistent level regardless of experimental conditions or cell/tissue types under study. For example, actin is described to decrease during adipogenesis, and as such would not be an applicable internal control in such studies [65]. We show herein that, overall, our differential gene expression data for EP *vs*. SC WAT depot at the cell and tissue level is of a similar magnitude when either Gapdh or acidic ribosomal phosphoprotein P0 (36B4) is used as an internal standard. This suggests that our findings are of a robust nature and not solely reflective of variation in expression of a single given internal control transcript across the analyzed samples.

While we do not at this time know the regulatory mechanisms behind the reduction of Mup transcript expression in *ob/ob *mice, it is of interest to note that reduced fertility occurs in the *ob/ob *mouse [66,67]. Mups are lipocalins that function as pheromones, either alone or when bound to small hydrophobic molecules [68,69] and are important in reproductive cycle of rodents where urine-derived signals control sexual attraction, mating and puberty onset [70-73]. As pheromones, Mup proteins control mating behavior and puberty onset in mice; reduced Mup transcript levels in *ob/ob *mice may conceivably be related to their infertility phenotype [66,67]. In this regard Mup expression, at least in mice, may be a molecular avenue whereby fat mass or fat distribution might impact mating and fertility. While it is unfortunate that the nearly similar sequence of a number of the Mup genes precludes a precise gene-by-gene analysis of each individual Mup transcript in this complex multigene family [48-57], nonetheless future studies on the nature and adipose depot specificity of the Mup gene(s) promoter regions may allow a more precise mapping and understanding of Mup gene expression and regulation in distinct WAT depots. There are no known close functional or sequence analogs of Mups in humans, with the odorant binding proteins the most closely related human proteins [74,75]. However, several lipocalin family members play roles in murine and/or human adipose tissues. For example, lipocalin-2, also known as neutrophil gelatinase associated lipocalin (NGAL) transcript and protein increases during *in vitro *adipogenesis of 3T3-L1 preadipocytes and is abundantly expressed in adipose tissue [76-78]. Circulating lipocalin-2 concentrations positively correlate with adipocyte mass, hypertriglyceridemia, hyperglycemia and insulin resistance [78-80]. The lipocalin retinol binding protein 4 (RBP4) has recently been reported to be a marker for abdominal fat mass in humans [81] and some studies have suggested a role for RBP4 in the pathogenesis of type 2 diabetes [82-87].

In the case of EP WAT, we find Boc transcript to show differential enrichment in EP *vs*. SC WAT when compared in purified adipocytes, stromal vascular fraction, as well as in intact WAT depots. Boc acts as a receptor for sonic hedgehog and is important for the guidance of commissural axons [88]. The Cdon and Boc complex also mediates cell-cell interactions between muscle precursors to promote myogenesis [89]. Hedgehog signaling is a very early event in the onset of adipogenesis [33]. Since myocytes and adipocytes are believed to share the same mesodermal progenitor cell type [90] and the hedgehog signaling pathway has been demonstrated to have an important role in fat formation [31-33], it is possible that Boc is involved in adipogenesis and/or adipocyte function. To our knowledge our findings on Boc transcript expression in WAT depots and the upregulation of Boc transcript in *ob/ob *WAT are the first to suggest a role for Boc in adipose tissue. At the least, our observations indicate that the function of Boc, and possibly its binding partner Cdon, should be considered in models that address the role of the hedgehog pathway in adipose tissue.

## Conclusion

It is possible that additional dissection of the mechanisms underlying the enrichment of Mup transcripts in the SC WAT depot and Boc transcript in the EP WAT depot may lead to novel insights on the molecular mechanisms governing gene expression in distinct WAT depots, for which very little knowledge currently exists. Studies along these lines may ultimately, for example, result in the design of promoter constructs that would allow for transgenesis or knockout studies to be conducted in a WAT depot-dependent manner. Future analyses of the transcriptional control of WAT depot specific gene regulation may also lead to key insights into regional adiposity and pinpoint WAT depot-specific therapeutic intervention targets in the fight against obesity and its complications.

## Abbreviations

FCS: fetal calf serum; PCR: polymerase chain reaction; qPCR: quantitative polymerase chain reaction; WAT: white adipose tissue; BAT: brown adipose tissue; SC: subcutaneous; EP: epididymal; RP: retroperitoneal; SCD: stearoyl CoA desaturase; aFABP: adipocyte fatty acid binding protein; MIX: methylisobutylxanthine; SSH: suppressive subtractive hybridization; Mup: major urinary protein; Boc: brother of Cdon.

## Authors' contributions

YW conducted all qPCR studies and analysis excepting the tissue expression. JYK prepared and screened the SSH library and did initial assessment of Mup transcript level by Northern blots. SZ conducted qPCR for tissue specific expression. CMS wrote the text, oversaw all experiments, and was responsible for planning and data analysis at all phases of the project. All authors read and approved the final manuscript.

## Supplementary Material

Additional file 1 Additional transcripts enriched in EP Adipocytes vs. SC Adipocytes Click here for file
