# Supplementary figures and images for: Differential screening identifies transcripts with depot-dependent expression in white adipose tissues
Source: BMC Genomics. 2008 Aug 22;9:397. doi: 10.1186/1471-2164-9-397 (PMC2547859; doi:10.1186/1471-2164-9-397)

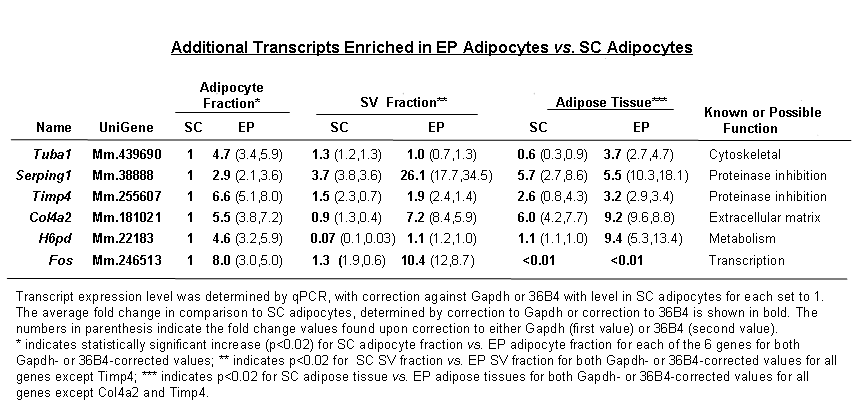

Supplement: Additional file 1 — Additional transcripts enriched in EP Adipocytes vs. SC Adipocytes [file 1471-2164-9-397-S1.tiff]
